# Predictors of Recovery from Traumatic Brain Injury-Induced Prolonged Consciousness Disorder

**DOI:** 10.1155/2017/9358092

**Published:** 2017-02-23

**Authors:** Hiroaki Abe, Keigo Shimoji, Yoshihide Nagamine, Satoru Fujiwara, Shin-Ichi Izumi

**Affiliations:** ^1^Department of Rehabilitation Medicine, Kohnan Hospital, Tohoku Ryogo Center, Sendai, Japan; ^2^Department of Physical Medicine and Rehabilitation, Graduate School of Medicine, Tohoku University, Sendai, Japan; ^3^Department of Diagnostic Radiology, Tokyo Metropolitan Geriatric Hospital, Tokyo, Japan; ^4^Department of Neurosurgery, Kohnan Hospital, Tohoku Ryogo Center, Sendai, Japan; ^5^Department of Neurosurgery, Kohnan Hospital, Sendai, Japan; ^6^Graduate School of Biomedical Engineering, Tohoku University, Sendai, Japan

## Abstract

We investigated the clinical predictors of the degree of recovery in patients with prolonged disorders of consciousness (PDC) caused by traumatic brain injury. Fourteen patients with PDC underwent two diffusion tensor imaging (DTI) studies; the first and second scans were performed at 345.6 ± 192.6 and 689.1 ± 272.2 days after the injury, respectively. In addition to the temporal changes in each of these diffusion parameters, fractional anisotropy (FA), mean diffusivity, axial diffusivity (AD), and radial diffusivity were assessed over a 1-year period. Relationship of clinical and DTI parameters with recovery from PDC (RPDC) was evaluated using Spearman's rank-correlation and stepwise multiple linear regression analysis. The mean FA and number of voxels with FA values > 0.4 (VsFA0.4) were significantly decreased at the second scan. A significant positive correlation was observed between the degree of RPDC and mean FA (*r* = 0.60) and VsFA0.4 (*r* = 0.68) as well as between the difference in VsFA0.4 (*r* = 0.63) and AD (*r* = 0.54) between the first and second scans. On multiple linear regression analysis, initial severity of PDC and the difference in AD remained significantly associated with the degree of RPDC. The microstructural white matter changes observed in this study indicate their potential relation with the degree of RPDC over the longer term.

## 1. Introduction 

Diffuse axonal injury (DAI) is a common form of traumatic brain injury (TBI) sustained in motor vehicle collisions. The injury involves rotational forces and is characterized by extensive white matter damage [1]. The neuropathology of disorders of consciousness (coma, vegetative state, and minimally conscious state) has been extensively described at postmortem [2–4]. Diffuse disruption of subcortical white matter is the most common postmortem finding in victims of TBI associated with impaired consciousness [2]. DAI and thalamic damage were the most common postmortem structural abnormalities reported in a case series of 35 patients who remained in a vegetative state after TBI until the time of their death [3, 4]. Thus, severe TBI commonly involves multiple diffuse lesions in both white and gray matter.

Diffusion tensor imaging (DTI) has been useful in describing the microstructure changes in the chronic stage of DAI [5]. Several studies have documented posttraumatic findings of axotomy and demyelination, months to years after injury. These include findings of increased water diffusion as measured by mean diffusivity (MD) and a reduction in the directionality of diffusion as measured by fractional anisotropy (FA) on DTI [5]. These findings, in conjunction with the apparent loss of subcortical white matter volume, suggest that acute edema may be a potential early marker of posttraumatic deterioration that ultimately impairs axonal integrity in the chronic state [5]. Few longitudinal studies have examined temporal evolution of white matter damage after DAI in humans [6–9]. However, the acute scans were collected, on average, within two months after injury; smaller time windows are necessary to obtain a more thorough understanding of the evolution of white matter damage. Further, several studies have documented correlation between decreased FA in several brain regions and unfavorable outcomes in patients with severe TBI [8, 10, 11]. A recent whole brain WM analysis of DTI parameters revealed an increase in axial diffusivity (AD) and radial diffusivity (RD) in the acute phase and a positive correlation of RD with severity of injury [5]. Longitudinal analysis showed reduction in FA and AD, but not in RD [5]. This study [5] examined the evolution of white matter integrity from acute to chronic stages of DAI due to TBI. Data from this study suggest complicated mild-to-severe TAI results in significant edema that eventually resolves leaving behind a compromised white matter microstructure. The data [5] also suggest that white matter compromise after DAI is a process involving white matter demyelination as well as axonal damage that may be present not only in the early stage but also in the chronic stage [5]. However, the mechanism that explains this finding is still unclear. DTI still appears to be capable of detecting microstructural changes after DAI.

Patients with severe PDC, such as those in a vegetative state, typically have unfavorable outcomes although gradual, subtle, and minor clinical changes are on record. In the last 2 years, our institution provided inpatient care to patients with prolonged disorders of consciousness (PDC) due to TBI. The therapeutic modalities include standard nursing care, physical therapy, and occupational therapy, as well as other alternative treatment modalities such as music therapy, aroma massage, and exposure to natural environment (i.e., feeling the sunlight, blowing wind, and exposure to seasonal temperature changes). Indeed, a few patients have shown slight positive reactions during inpatient residency at our institution.

In this study, we sought to identify potential predictors of the degree of recovery from PDC over a period of 2 years, using DTI parameters in whole brain. We examined the longitudinal alterations in anatomical connections of white matter in these patients and explored potential association of microstructural imaging biomarkers in whole brain white and gray matter with clinical markers of potential clinical relevance.

## 2. Methods

### 2.1. Patients

We retrospectively recruited 14 patients at our institution that had chronic severe PDC resulting from traffic accident-related brain injury. All patients with PDC underwent 3 T MRI studies at admission and after completion of 1 year of treatment at our institution. Mean age (±SD) of patients was 58.6 ± 19.3 years; the mean duration (±SD) from admission to study recruitment was 339.8 ± 191.7 days. The clinical characteristics of patients are presented in Table 1. The first DTI scan was performed at admission (345.6 ± 192.6 days after the initial injury); the second scan was performed approximately 1 year after admission (689.0 ± 272.2 days after the initial injury).

Furthermore, we prospectively recruited 8 healthy normal volunteers (2 males and 6 females) for comparison. The age range of the healthy participants was 32–60 (45.5 ± 9.1) years (Table 2).

### 2.2. Standard Protocol Approvals, Registrations, and Patient Consent

This study was conducted in compliance with the ethical principles for biomedical research on human subjects enshrined in the Declaration of Helsinki and informed consent regulations. Approval from the institutional ethics committee was obtained prior to the initiation of the study.

### 2.3. PDC Assessment

PDC was assessed using the Kohnan score, which measures the severity of consciousness disorder from a severe, persistent vegetative state (Appendix [12]). The Kohnan score was developed at our institution to resolve this issue. The unidimensionality and higher intra- and interrater reliability of this score have previously been reported [12]. The score is based on seven parameters, each of which is subdivided into 5 grades: extreme (10 points), severe (9 points), moderate (7 or 8 points), mild (5 points), and slight (0 points). We additionally assessed general functional recovery using the Extended Glasgow Outcome Scale (GOSE) (range 1–8; higher scores indicate superior functional outcomes). These assessments were performed at admission and at 2 years after admission.

### 2.4. Image Acquisition

MRI was performed using a 3.0 T Signa Excite HD scanner (General Electric, Milwaukee, WI, USA). The parameters for DTI were as follows: echo time, 59 ms; repetition time, 9,000 ms; flip angle, 90°; slice thickness, 3 mm with no gap; field of view, 28.8 × 28.8 cm; acquisition matrix, 96 × 96; image matrix, 256 × 256 with a voxel size of 1.125 × 1.125 × 3.0 mm; number of excitations, 1; and band width, 250 kHz. Images were obtained using 15-directional diffusion encoding (*b* value, 1,000 s/mm^2^ in each direction) and one set of images with *b* = 0 s/mm^2^.

A total of 46 axial sections covering the entire cerebrum were obtained. The most inferior DTI slices were positioned at the medulla oblongata during acquisition.

### 2.5. Image Preprocessing

All DTI data were processed using the FSL software package (the Oxford Centre for Functional Magnetic Resonance Imaging of the Brain Software Library [13]). DTI images were skull-stripped using the Brain Extraction Tool (BET) in the FSL. The images were corrected using the Eddy Correct program of the FSL package to adjust for the effects of head movement and eddy currents. FA, MD, AD, and RD maps were generated using dtifit program in the FSL. Lastly, we calculated the mean FA, MD, AD, and RD and counted the number of voxels in which FA values were >0.4 in the whole brain (VsFA0.4) using fslstats program implemented in the FSL. We assumed that using whole brain method affected not only white matter changes but also gray matter and ventricles. To eliminate these effects, we chose the method of VsFA0.4 that selects only the voxel with high FA values.

### 2.6. Statistical Analysis

Statistical analyses were performed using the Statistical Package for the Social Sciences (SPSS) 24.0 for Mac (IBM SPSS, Chicago, IL, USA). Comparisons over time for group Kohnan scores, GOSE, FA, VsFA0.4, MD, AD, and RD were conducted using the paired *t*-test or Wilcoxon rank-sum test according to the results of the Shapiro–Wilk test. The Spearman rank-correlation coefficient was used to evaluate the relationships between age, time of admission from injury, degree of recovery from PDC (as determined by change in the Kohnan score during the 2-year period after admission), and the diffusion tensor parameters. These diffusion tensor parameters used for comparisons were FA, VsFA0.4 MD, AD, and RD in the first scan and the differences in the same parameters between the first and the second scan. All univariate potential factors with a *P* value < 0.1 were entered into the multivariate linear regression model. In addition, variance inflation factors were computed to examine the possible collinearity problem among the predictors. Stepwise multiple regression analyses were performed to identify the variables associated with the degree of recovery from PDC. The alpha level was set to 0.05 for all statistical tests and adjusted for multiple tests (e.g., *n* = 5, *P* > 0.01, comparison of the mean FA, MD, AD, and RD) with Bonferroni correction.

## 3. Results

In this study, 12 patients had a GOSE score of 2 (vegetative state), and 2 patients had GOSE score of 3 (severe disorder) at admission. However, Kohnan scores at admission showed more variability (range: 29–68). These ranged from minimum consciousness disorder (<39) to complete vegetative state (>65). After 2 years, there was no significant change in GOSE scores (*P* = 0.16, Wilcoxon rank-sum test): 10 patients had a GOSE score of 2, while 4 patients had a GOSE score of 3. In contrast, a subset of PDC patients showed a significant recovery based on the Kohnan score (58.9 ± 12.3* versus *48.7 ± 22.7, *P* < 0.01; Wilcoxon rank-sum test) (Table 3). The mean difference in Kohnan scores at admission and at completion of 2 years was 10.14 ± 11.10.

In patients with PDC, FA, VsFA0.4, MD, AD, and RD values in the first scan were 0.24 ± 0.03, 43715. 14 ± 20913.54, 1.47 ± 0.15 [×10^−3^ mm^2^/s], 1.82 ± 0.18 [×10^−3 ^mm^2^/s], and 1.30 ± 0.14 [×10^−3 ^mm^2^/s], respectively, whereas those in the second scan were 0.22 ± 0.02, 33036.07 ± 10413.59, 1.47 ± 0.01 [×10^−3 ^mm^2^/s], 1.79 ± 0.15 [×10^−3^ mm^2^/s], and 1.31 ± 0.13 [×10^−3 ^mm^2^/s], respectively (Table 3). Patients with PDC showed significant changes in FA (*P* = 0.006, paired *t*-test) and VsFA0.4 (*P* = 0.003, Wilcoxon rank-sum test) over time. However, there were no significant differences in MD (*P* = 0.993, paired *t*-test), AD (*P* = 0.506, paired *t*-test), and RD (*P* = 0.650, paired *t*-test) values between the first and second scans (Table 4). In contrast, any significant change of DTI parameters was not shown in the 8 normal healthy participants (Table 5).

No significant correlations were observed between the degree of recovery from PDC and age. Kohnan score was strongly associated (*r* = −0.76, *P* = 0.002) with the degree of recovery from PDC. Among the DTI parameters, significant correlations were observed between the degree of recovery from PDC and FA (*r* = 0.60, *P* < 0.05) and VsFA0.4 at the first scan (*r* = 0.68, *P* = 0.008); however, no significant correlation was observed between the MD, AD, and LD at the first scan (Table 6). In the longitudinal DTI parameters, no significant correlations were observed between the degree of recovery from PDC and difference in FA, MD, and RD over time (Table 6). However, a significant positive correlation was observed between the degree of recovery from PDC and time-dependent changes in AD (*r* = 0.544, *P* < 0.05) and VsFA0.4 (*r* = 0.624, *P* < 0.05) between the first and second scans (Table 6).

Variables showing a significant association (*P* value < 0.1) were included in the Spearman correlation analysis. Thus, day of institution admission from injury, Kohnan score at the first assessment, FA at the first scan, VsFA0.4 at the first scan, difference in VsFA0.4, and MD and AD from the first to the second scan were included in the multivariate regression analysis. Stepwise multiple regression analysis revealed that the Kohnan score at the first scan (standardized *β* = −0.723, 95% CI: 32.7–63.0, *P* < 0.0001) and difference in AD over time (standardized *β* = 0.337, 95% CI: 4610.7–46541.3, *P* < 0.05) accurately predicted the degree of recovery from PDC [adjusted *R*^2^ = 0.841].

## 4. Discussion

We explored potential clinical predictors of the degree of recovery from PDC after 2 years of admission, with a particular focus on the DTI parameters. Our results showed significantly decreased FA values and number of voxels in which FA values were >0.4 (VsFA0.4) in whole brain, but MD, AD, and RD showed no significant change. In contrast, healthy participants did not exhibit such changes. On correlation analysis, Kohnan score and FA and VsFA0.4, the difference of VsFA0.4, and AD were significantly correlated in monovariable analysis. Multiple regression analysis revealed that each Kohan score in the first assessment and the difference in AD over time have statistically significant contribution to the degree of recovery from PDC. Currently, to the best of our knowledge, there are no known predictors of long-term positive clinical response in patients with PDC. Several clinicians were forced to provide long-term care without established specific clinical goals for patients with PDC. Therefore, it is critical to develop predictors of long-term changes elicited through long-term therapeutic intervention in these patients. Our results demonstrate that microstructural white matter changes occurred in patients with PDC, which suggests that the assessment of white matter changes may help identify valid long-term outcome predictors in these patients.

DTI is based upon the diffusivity of water molecules, which varies in different tissues [14]. In white matter, it is more limited in the directions of diffusion. In healthy tracts, the anisotropy (limited directionality of diffusion) is higher than that in the gray matter. This difference allows for the calculation of fractional anisotropy (FA) values for tissue and the generation of white matter fiber maps. FA ranges from 0 to 1, where 0 represents isotropic diffusion (or lack of directional organization) and 1 represents anisotropic diffusion (or organized tissue such as white matter tracts) [15]. Studies have demonstrated the potential utility of DTI for providing quantitative assessments of microstructural damage in TBI, in which DAI is common [1–11, 14–16]. Although the specifics are still not well understood, FA is believed to be influenced by many factors, including the degree of myelination and axonal density and/or integrity [9, 17–19].

FA has been shown to decrease in patients with mild and moderate/severe brain injury [1, 4, 5, 11, 14–16, 19–28]. Perez et al. [5] reported significantly lower FA in the chronic phase of TBI as compared to that in healthy controls; in contrast, AD, MD, and RD in chronic patients were significantly higher than in healthy controls. Additionally, chronic FA showed a positive correlation with processing speed [28]. In this study, patients with chronic PDC had significantly lower FA and VsFA0.4 at 2 years as compared to those at admission. Our first scan data were collected 345.6 ± 192.6 days after injury, which corresponds to the chronic stage of white matter damage. Thus, our results suggest occurrence of microstructural change over the longer term. On univariate correlation analysis, FA and VsFA0.4 at the first scan significantly correlated with the degree of recovery of PDC, which is consistent with previous studies [5–8, 10, 11, 14, 20–29] where patients with higher FA showed good recovery. Temporal change in VsFA0.4 and AD significantly correlated with the degree of recovery from PDC. This probably indicates that minor microstructural changes correlate with the degree of recovery from PDC. Several previous studies [6, 8, 10, 11, 20, 25–29] have reported a positive association of FA with higher cognitive function and outcomes. However, in this study, higher difference of VsFA0.4, MD, and AD positively correlated with the degree of recovery from PDC. In other words, a time-dependent decrease in VsFA0.4, MD, and AD may be related to a better outcome in PDC. In this respect, our results are not inconsistent with those of several previous reports [6, 8, 10, 11, 20, 25–29], which indicated that a time-dependent decrease in FA was associated with poor outcomes as assessed by cognitive function and GOSE. These contradictory findings may reflect the difference in PDC severity between studies; most of our patients had GOSE scores of ≤3 and showed subtle and minor clinical changes. Although significant recoveries were observed based on Kohnan scores, the changes were not significant when based on GOSE scores, which suggests that improvement in our patients was lower than that reported in previous studies [6, 8, 10, 11, 20, 25–29]. Our subjects generally had more severe outcomes; all our patients had GOSE scores ≤ 3, which indicates more severe disability than that among patients in the previous studies [6, 8, 10, 11, 20, 25–29]. Thus, interstudy differences in results may reflect DAI severity. Differences with respect to time elapsed since injury may also have contributed to the divergent findings; we included patients with PDC in the extended chronic phase.

In patients with PDC, the degree of recovery was very small, as shown by the lack of change in GOSE scores. In our participants, induction of a severe inactive state due to brain injury might lead to secondary neurodegeneration that might be detected by DTI. However, the underlying mechanism behind longitudinal alterations in white matter remains unclear. In addition, this study had some limitations. The number of subjects was small, and they had various pathological states, such as contusion, subarachnoid hemorrhage, and intracranial hemorrhage. Thus, further longitudinal studies are warranted that combine DTI with volumetric measurements and other detailed analyses, such as functional connectivity analysis. The potential of DTI use as a prognostic tool needs further investigation in studies with a larger number of subjects.

In conclusion, on stepwise multiple linear regression analysis, Kohnan score and the difference in AD showed a significant association with the degree of recovery from PDC. In other words, we demonstrate evidence of temporal microstructural white matter changes in patients with PDC; DTI parameters are useful indices for assessment of white matter alterations in these patients. As a noninvasive modality, DTI provides in vivo quantitative pathophysiological information. Tracking white matter microstructural changes over time has the potential to measure neuroplasticity and repair after TBI and may eventually be utilized to monitor therapeutic responses. Further research is required to investigate the leads identified in this study.

## Figures and Tables

**Table 1:** Clinical profiles of patients with prolonged consciousness disorders due to brain injury.

	Age (y)	Gender	Diagnosis	Days until admission following injury	Days until the first scan from injury	Days until the second scan from injury	GCSE at admission	GCSE after 1 year from admission	Kohnan score at admission	Kohnan score after 1 year from admission	Degree of improvement from prolonged consciousness disorders^*∗*^
1	75	Female	Right acute subdural hematoma, cerebral contusion	407	415	768	2	2	65	63	2
2	60	Male	Traumatic subarachnoid hemorrhage, pneumocephalus, diffuse axonal injury	264	266	548	3	3	34	5	29
3	68	Male	Traumatic subarachnoid hemorrhage, diffuse axonal injury, brainstem contusion	296	311	636	2	2	67	68	−1
4	68	Female	Cerebral contusion, traumatic subarachnoid hemorrhage, diffuse axonal injury, brainstem contusion	126	135	416	2	2	63	56	7
5	78	Female	Left subdural hematoma, traumatic subarachnoid hemorrhage, intracerebral hemorrhage	629	629	885	2	2	64	62	2
6	86	Male	Traumatic subarachnoid hemorrhage	155	158	591	2	2	68	64	4
7	44	Female	Traumatic subarachnoid hemorrhage, diffuse axonal injury	230	232	396	2	3	55	34	21
8	58	Male	Traumatic subarachnoid hemorrhage, left intracerebral hemorrhage	147	149	286	3	3	29	0	29
9	77	Female	Traumatic subarachnoid hemorrhage, cerebral contusion, left intracerebral hemorrhage	226	228	580	2	2	63	62	1
10	31	Male	Left intraventricular hemorrhage, left intracerebral hemorrhage, diffuse axonal injury	482	490	1173	2	2	67	64	3
11	56	Male	Cerebral contusion, intraventricular hemorrhage, brainstem contusion	350	360	779	2	2	62	52	10
12	64	Male	Traumatic subarachnoid hemorrhage, diffuse axonal injury, cerebral contusion	391	400	849	2	2	65	64	1
13	23	Female	Right acute subdural hematoma, traumatic subarachnoid hemorrhage, cerebral contusion, diffuse axonal injury	798	807	1200	2	2	67	65	2
14	33	Male	Traumatic subarachnoid hemorrhage, cerebral contusion, left cerebral hemorrhage	256	258	540	2	3	55	34	21

*∗* is the difference in Kohnan score from admission to after 1 year.

**Table 2:** Each diffusion parameter of patients with prolonged consciousness disorders due to brain injury.

Number	FA at the first scan	FA at the second scan	Difference in FA from the first to the second scan	VsFA0.4 at the first scan	VsFA0.4 at the second scan	Difference in VsFA0.4 from the first to the second scan	MD [×10^−3^ mm^2^/s] at the first scan	MD [×10^−3^ mm^2^/s] at the second scan	Difference in MD [×10^−3^ mm^2^/s] from the first to the second scan	AD [×10^−3^ mm^2^/s] at the first scan	AD [×10^−3^ mm^2^/s] at the second scan	Difference in AD [×10^−3^ mm^2^/s] from the first to the second scan	RD [×10^−3^ mm^2^/s] at the first scan	RD [×10^−3^ mm^2^/s] at the second scan	Difference in RD [×10^−3^ mm^2^/s] from the first to the second scan
1	0.21	0.21	0.00	21895	23414	−1519	1.52	1.66	−0.14	1.83	2.00	−0.17	1.37	1.49	−0.12
2	0.24	0.22	0.01	47948	39756	8192	1.48	1.40	0.08	1.82	1.71	0.11	1.31	1.25	0.06
3	0.22	0.21	0.01	35733	30750	4983	1.42	1.46	−0.04	1.74	1.77	−0.03	1.26	1.30	−0.05
4	0.22	0.21	0.01	30267	26690	3577	1.36	1.41	−0.05	1.66	1.71	−0.05	1.22	1.27	−0.05
5	0.22	0.22	0.00	31767	30802	965	1.56	1.62	−0.06	1.90	1.97	−0.07	1.39	1.44	−0.05
6	0.29	0.25	0.04	77478	55501	21977	1.43	1.52	−0.09	1.84	1.89	−0.06	1.23	1.33	−0.10
7	0.24	0.22	0.02	37712	29094	8618	1.17	1.18	−0.02	1.48	1.46	0.02	1.01	1.05	−0.04
8	0.28	0.21	0.07	84429	39949	44480	1.39	1.38	0.02	1.81	1.67	0.14	1.19	1.23	−0.04
9	0.21	0.21	0.00	24037	26024	−1987	1.67	1.66	0.01	2.02	2.00	0.01	1.49	1.49	0.01
10	0.19	0.19	0.00	28011	21844	6167	1.47	1.54	−0.07	1.74	1.83	−0.09	1.33	1.39	−0.07
11	0.29	0.24	0.05	72447	40235	32212	1.39	1.30	0.09	1.80	1.62	0.18	1.18	1.14	0.05
12	0.22	0.21	0.01	37434	33854	3580	1.43	1.50	−0.07	1.74	1.80	−0.06	1.28	1.34	−0.07
13	0.22	0.20	0.03	26234	17710	8524	1.52	1.51	0.00	1.86	1.82	0.04	1.34	1.36	−0.02
14	0.26	0.25	0.00	56620	46882	9738	1.81	1.47	0.34	2.27	1.86	0.41	1.58	1.28	0.30

FA: fractional anisotropy; VsFA0.4: number of voxels with a fractional anisotropy value greater than 0.4 in the whole brain; MD: mean diffusivity; AD: axial diffusivity; RD: radial diffusivity.

**Table 3:** Each diffusion parameter in normal healthy participants.

Number	Age (years)	Gender	FA at the first scan	FA at the second scan	Difference in FA from the first to the second scan	VsFA0.4 at the first scan	VsFA0.4 at the second scan	Difference in VsFA0.4 from the first to the second scan	MD [×10^−3^ mm^2^/s] at the first scan	MD [×10^−3^ mm^2^/s] at the second scan	Difference in MD [×10^−3^ mm^2^/s] from the first to the second scan	AD [×10^−3^ mm^2^/s] at the first scan	AD [×10^−3^ mm^2^/s] at the second scan	Difference in AD [×10^−3^ mm^2^/s] from the first to the second scan	RD [×10^−3^ mm^2^/s] at the first scan	RD [×10^−3^ mm^2^/s] at the second scan	Difference in RD [×10^−3^ mm^2^/s] from the first to the second scan
1	60	Female	0.26	96457	1.00	1.25	0.88	0.26	97531	1.02	1.27	0.89	0.26	96457	1.00	1.25	0.88
2	49	Female	0.26	111993	1.05	1.31	0.92	0.26	115720	1.04	1.30	0.91	0.26	111993	1.05	1.31	0.92
3	44	Female	0.26	123996	1.01	1.26	0.89	0.26	125776	1.01	1.27	0.88	0.26	123996	1.01	1.26	0.89
4	43	Female	0.27	122068	1.01	1.28	0.88	0.27	125151	1.01	1.28	0.88	0.27	122068	1.01	1.28	0.88
5	54	Female	0.27	150473	0.96	1.22	0.83	0.28	149240	0.99	1.26	0.86	0.27	150473	0.96	1.22	0.83
6	46	Female	0.26	118282	0.98	1.23	0.85	0.27	122442	0.99	1.25	0.86	0.26	118282	0.98	1.23	0.85
7	32	Male	0.26	111993	1.05	1.31	0.92	0.29	135897	0.88	1.14	0.75	0.26	111993	1.05	1.31	0.92
8	36	Male	0.28	144917	0.99	1.26	0.86	0.27	145240	0.99	1.26	0.86	0.28	144917	0.99	1.26	0.86

FA: fractional anisotropy; VsFA0.4: number of voxels with a fractional anisotropy value greater than 0.4 in the whole brain; MD: mean diffusivity; AD: axial diffusivity; RD: radial diffusivity.

**Table 4:** Comparison of diffusion tensor parameters and consciousness disorders.

Diffusion parameters	First scan		Second scan		*P* values
	Mean	Standard deviation	Mean	Standard deviation	
Kohnan score^*∗*^	58.86	12.30	48.71	22.69	0.001
GSCE^*∗*^	2.14	0.36	2.29	0.47	0.157
Fractional anisotropy	0.24	0.03	0.22	0.02	0.006
Number of voxels with a fractional anisotropy value greater than 0.4 in the whole brain^*∗*^	43715.14	20913.55	33036.07	10413.59	0.003
Mean diffusivity (MD) [×10^−3^ mm^2^/s]	1.47	0.15	1.47	0.01	0.993
Axial diffusivity (AD) [×10^−3^ mm^2^/s]	1.82	0.18	1.79	0.15	0.506
Radial diffusivity (RD) [×10^−3^ mm^2^/s]	1.30	0.14	1.31	0.13	0.65

^*∗*^Wilcoxon rank test.

**Table 5:** Comparison of diffusion tensor parameters in normal healthy participants.

Diffusion parameters	First scan		Second scan		*P* values
	Mean	Standard deviation	Mean	Standard deviation	
Fractional anisotropy	0.26	0.07	0.27	0.01	0.13
Number of voxels with a fractional anisotropy value greater than 0.4 in the whole brain	122522.38	17745.24	127124.63	16594.75	0.15
Mean diffusivity (MD) [×10^−3^ mm^2^/s]^*∗*^	1.01	0.03	0.99	0.05	0.40
Axial diffusivity (AD) [×10^−3^ mm^2^/s]^*∗*^	1.26	0.03	1.25	0.05	0.23
Radial diffusivity (RD) [×10^−3^ mm^2^/s]^*∗*^	0.88	0.03	0.09	0.05	0.57

^*∗*^Wilcoxon rank test.

**Table 6:** Correlation between the degree of recovery from prolonged consciousness disorder and each value.

Variable	Degree of recovery from PCD	
	*r* (Spearman correlation coefficient)	*P* values
At the first scan or assessment		
Age	−0.24	0.41
Day of institution admission from injury	−0.48	0.086
Kohnan score	−0.76	0.002
Fractional anisotropy	0.60	0.023
Numbers of voxels with a fractional anisotropy value greater than 0.4	0.68	0.008
Mean diffusivity	−0.22	0.459
Axial diffusivity	−0.01	0.976
Radial diffusivity	−0.34	0.241
The difference between the first and the second scan		
Fractional anisotropy	0.38	0.177
Numbers of voxels with a fractional anisotropy value greater than 0.4	0.62	0.017
Mean diffusivity	0.48	0.086
Axial diffusivity	0.54	0.044
Radial diffusivity	0.40	0.162

**Table 7:** 

Clinical symptoms	Grade				
	Extreme (10)	Severe (9)	Moderate (8/7)	Mild (5)	Slight (0)
Voluntary movement	(1) Absent (2) Acrocontracture (3) Pain reflex but slight trembling and rough breathing	(1) Almost absent but parts of the extremities move minutely (2) Part of the extremity flexed and part paralyzed (3) Pain reflex or no pain reflex with clearly frowning face	(1) Occasional all/partial extremity movement with no intention (2) Extremity could be paretic (3) Brushing away reaction for pain	(1) Occasional movement to meet an object (2) Capable of raising the arms upward or moving them in the intended direction, that is, face or head, imitating a posture of the tester	(1) Capable of movement with intention (2) Capable of unassisted posture change (partial change inclusive) (3) Moving wheelchair unassisted, even if awkwardly

Voluntary ingestion	Totally incapable of masticating and swallowing; on tube nutrition (gastric/nasal feeding)	(1) Almost on tube nutrition (2) Saliva swallowing or mastication is found (3) Capable of attempting slight perusal ingestion, that is, fruit juice, custard pudding, and so forth	(1) Capable of masticating; even if not, almost capable of assisted auroral ingestion by swallowing, though sometimes choking (2) Insufficient peroral ingestion requires tube nutrition	(1) Capable of unassisted ingestion by swallowing; mastication could be awkward (2) Capable of ingesting all the rice gruel served or chopped food with assistance (3) Attempting to reach mouth with a passed spoon or put the food into mouth awkwardly	Ingesting on own using spoon awkwardly

Fecal and urinary incontinence	No observed somatic movement when evacuating/urinating	Slight somatic movement when evacuating/urinating	After incontinence, a displeased look or some signal is observed, that is, frequent somatic movement	(1) Forced regular evacuating and urinating lead to the prevention of fecal and urinary incontinence (2) Communicating the fact in a certain way after incontinence	Except during the night, preevacuation and preurination communication is possible

Ophthalmography and visual recognition	(1) Eyes not opened (2) Eyes opened, no blink reflex	(1) Eyes opened, blink reflex (2) No following ocular movement and no focusing eyes on an object	(1) Looking straight toward the direction of the call (2) Following a moving object or staring at a TV, although understanding is impossible	(1) Discriminating close relatives followed by a facial expression (2) Favorite picture, among other things, induces a facial expression	(1) Capable of reading easy words (2) Capable of understanding simple numbers (3) When watching TV, response and laughter are apparent

Vocalizing and utterances	(1) No vocalizing (2) No lip movement under tracheostomy	(1) Groaning, among others, without meaningful utterances (2) Lip movement observed under tracheostomy	(1) A short utterance though not understandable (2) Occasional inarticulate vocal response to calls (3) Under tracheostomy, response to calls is through lip movement	(1) Occasional vocalizing of a meaningful word (2) Vocal response to calls (3) Imitating talking by the tester under tracheostomy	(1) Capable of vocalizing a simple word response (2) Lip movement corresponds to what is asked

Change of expression	No response to ambient sound stimulations and TV sounds, among other things	Change of expression, such as smiling, crying, and anger, is not due to ambient stimulations	Change of expression is occasionally found in response to ambient stimulations	Change of expression, such as smiling, crying, and anger, closely matches an expected response to the ambient stimulation	Change of expression, such as crying and smiling, exactly matches an expected response to the ambient stimulation

## Appendix

See Table 7.
