# Corrigendum to “Predictors of Recovery from Traumatic Brain Injury-Induced Prolonged Consciousness Disorder”

**DOI:** 10.1155/2020/7169025

**Published:** 2020-11-04

**Authors:** Hiroaki Abe, Keigo Shimoji, Yoshihide Nagamine, Satoru Fujiwara, Shin-Ichi Izumi

**Affiliations:** ^1^Department of Rehabilitation Medicine, Kohnan Hospital, Tohoku Ryogo Center, Sendai, Japan; ^2^Department of Physical Medicine and Rehabilitation, Graduate School of Medicine, Tohoku University, Sendai, Japan; ^3^Department of Diagnostic Radiology, Tokyo Metropolitan Geriatric Hospital, Tokyo, Japan; ^4^Department of Neurosurgery, Kohnan Hospital, Tohoku Ryogo Center, Sendai, Japan; ^5^Department of Neurosurgery, Kohnan Hospital, Sendai, Japan; ^6^Graduate School of Biomedical Engineering, Tohoku University, Sendai, Japan

In the article titled “Predictors of Recovery from Traumatic Brain Injury-Induced Prolonged Consciousness Disorder” [1], one subscore of the Kohnan score was omitted from the appendix in error. The correct Kohnan score should compose of 7 subscores, and the corrected appendix is as follows:

## Figures and Tables

**Table 7:** 

	Grade				
Clinical symptoms	Extreme (10)	Severe (9)	Moderate (8/7)	Mild (5)	Slight (0)
Voluntary movement	(1) Absent (2) Acrocontracture (3) Pain reflex but slight trembling and rough breathing	(1) Almost absent but parts of the extremities move minutely (2) Part of the extremity flexed and part paralyzed (3) Pain reflex or no pain reflex with clearly frowning face	(1) Occasional all/partial extremity movement with no intention (2) Extremity could be paretic (3) Brushing away reaction for pain	(1) Occasional movement to meet an object (2) Capable of raising the arms upward or moving them in the intended direction, that is, face or head, imitating a posture of the tester	(1) Capable of movement with intention (2) Capable of unassisted posture change (partial change inclusive) (3) Moving wheelchair unassisted, even if awkwardly
Voluntary ingestion	Totally incapable of masticating and swallowing; on tube nutrition (gastric/nasal feeding)	(1) Almost on tube nutrition (2) Saliva swallowing or mastication is found (3) Capable of attempting slight perusal ingestion, that is, fruit juice, custard pudding, and so forth	(1) Capable of masticating; even if not, almost capable of assisted peroral ingestion by swallowing, though sometimes choking (2) Insufficient peroral ingestion requires tube nutrition	(1) Capable of unassisted ingestion by swallowing; mastication could be awkward (2) Capable of ingesting all the rice gruel served or chopped food with assistance (3) Attempting to reach the mouth with a passed spoon or put the food into the mouth awkwardly	Ingesting on own using spoon awkwardly
Fecal and urinary incontinence	No observed somatic movement when evacuating/urinating	Slight somatic movement when evacuating/urinating	After incontinence, a displeased look or some signal is observed, that is, frequent somatic movement	(1) Forced regular evacuating and urinating leads to the prevention of fecal and urinary incontinence (2) Communicating the fact in a certain way after incontinence	Except during the night, preevacuation and preurination communication is possible
Ophthalmography and visual recognition	(1) Eyes not opened (2) Eyes opened, no blink reflex	(1) Eyes opened, blink reflex (2) No following ocular movement, and no focusing eyes on an object	(1) Looking straight toward the direction of the call (2) Following a moving object or staring at a TV, although understanding is impossible	(1) Discriminating close relatives followed by a facial expression (2) Favorite picture, among other things, induce a facial expression	(1) Capable of reading easy words (2) Capable of understanding simple numbers (3) When watching TV, response and laughter are apparent
Vocalizing and utterances	(1) No vocalizing (2) No lip movement under tracheostomy	(1) Groaning etc., without meaningful utterances (2) Lip movement observed under tracheostomy	(1) A short utterance though not understandable (2) Occasional inarticulate vocal response to calls (3) Under tracheostomy, response to calls is through lip movement	(1) Occasional vocalizing of a meaningful word (2) Vocal response to calls (3) Imitating talking by the tester under tracheostomy	(1) Capable of vocalizing a simple word response (2) Lip movement corresponds to what is asked
Response and comprehension	No response to calls	Some response to calls, such as somatic or eye movement, etc.	Response to calls is possible at times, but no understanding	Response and understanding of simple calls is possible at times	Response fits the purpose of calls and nearly correct understanding
Change of expression	No response to ambient sound stimulations and TV sounds, etc.	Change of expression, such as smiling, crying, and anger, is not due to ambient stimulations	Change of expression is occasionally found in response to ambient stimulations	Change of expression, such as smiling, crying and anger closely matches an expected response to the ambient stimulation	Change of expression, such as crying and smiling, exactly matches an expected response to the ambient stimulation

## References

[1] Abe H., Shimoji K., Nagamine Y., Fujiwara S., Izumi S.-I. (2017). Predictors of recovery from traumatic brain injury-induced prolonged consciousness disorder. *Neural Plasticity*.

